# Evolving Business Models in Orthotics

**DOI:** 10.33137/cpoj.v4i2.35876

**Published:** 2021-09-21

**Authors:** N Schneider

**Affiliations:** Braceworks Custom Orthotics, 1-3500 24 Ave NW, Calgary, Alberta, Canada.

**Keywords:** Health Economics, Orthotics, Business Models, Rehabilitation, Alberta Aids to Daily Living, Funding

## Abstract

This submission provides an important historical context for understanding the current challenge facing the Orthotic and Prosthetic community in Alberta including Alberta Aids to Daily Living (AADL), Suppliers, and Providers: maintaining sustainable access to Orthotic care for people with mobility disorders in the face of declining real rates of reimbursement combined with increasing costs and a shortage of skilled Clinicians. Under the Canada Health Act, the federal government delegates responsibility for providing health care to the provinces. This delegation of responsibility to the provinces results in a degree of variability of funding of Orthotics and Prosthetics between provinces across the country. Funding of Orthotics and Prosthetics in Alberta is characterized by structural inequities that favour Prosthetics at the expense of Orthotics. To the extent that the structural inequities that exist in Alberta are related to governance by volunteer-run, non-profit organizations, they may be generalized to the Canadian experience. Finally, in a Call to Action a number of recommendations are made to address the challenge of sustainable access to Orthotic care in Alberta serving as a model for other provinces across Canada.

## INTRODUCTION

Braceworks specializes in orthotic treatment for children with neuromuscular-skeletal disorders. The clinical practice of pediatric orthotics is informed by applied research and development (R&D). The clinic is located at the University of Calgary in the hub of the local medical/research community enabling ease of collaboration with the Schulich School of Engineering and the Alberta Children's Hospital. In particular, Braceworks is engaged in developing an objective and quantifiable approach to the assessment and treatment of chest wall deformities. The current research is focused on Novel 3D imaging for chest wall anomalies: The early Calgary experience.^1^ This builds on previous research including The Calgary Protocol for bracing Pectus Carinatum: A Preliminary Report^2^ and Bracing of Pectus Carinatum: A Quantitative Analysis.^3^ Braceworks' specific contributions to the research include clinical knowledge, insight and experience, recruitment of study participants, data collection and analysis, and direct and indirect funding. AADL was established in 1980 to assist Albertans with a long-term disability, chronic illness or terminal illness, in maintaining independence in their community through the provision of basic medical equipment and supplies to meet clinically assessed needs. Sustainable access to orthotic care in Alberta faces significant economic challenges. As presented at a meeting of the Alberta Association of Orthotists and Prosthetists (AAOP) by Dr. Philip Jacobs (May 17, 2001), these challenges include: a retail model of pricing of procedures that rewards Prosthetics at the expense of Orthotics, a shortage of skilled orthotists identified in the Canadian P&O demographic study 2011,^4^ a labour market distorted by a public sector premium for prosthetic and orthotic technicians^5^ and clinicians,^6^ and a lack of success of orthotists to mature into a licensed profession regulated under the Alberta Health Professions Act.^7^

### Pricing of Orthotic Procedures in Alberta

The current AADL approved product lists – orthotics^8^ and prosthetics^9^ is loosely based upon the “Cost accounting manual–a step-by-step guide to an effective cost accounting system for the orthotic and prosthetic facility” developed by the American Orthotics and Prosthetics Association (AOPA), with AAOP making two significant modifications to the AOPA cost accounting system upon implementation by AADL in 1991:

1. AAOP discounted all the times to perform orthotic procedures in the AOPA cost accounting system by 20%, as reported at an AAOP Meeting by Mr. David Moe (September 19, 2002).
2. AAOP introduced a profit margin on material costs, and a profit margin that varied from component-to-component. The original AOPA cost accounting system contains no profit margin on material costs.

In 2001, AADL engaged Dr. Philip Jacobs representing the Institute of Health Economics of Edmonton to review the AAOP version of the “cost accounting system”. In his report, Dr. Jacobs concluded that the component-based formula was needlessly complex and contained some peculiar incentives related to markup on components.

Dr. Jacobs proposed two new formulas intended to simplify the existing formula and remove the inequities that rewarded high component and low labour practices (i.e. prosthetics) at the expense of low component and high labour practices (i.e. orthotics). He proposed a service model based on times to perform procedures for Orthotics and a retail model based on markup on components for Prosthetics:


Price=direct materials+(rate×time)

In response to Dr. Jacob's research, AAOP engaged Framework Partners Inc. of Calgary in October 2001 to review the AAOP version of the cost accounting system and Dr. Jacob's proposed formulas while undertaking a comprehensive survey to update the times to perform procedures.

It took considerable effort for all 28 Prosthetic and Orthotic Providers in Alberta to fully appreciate the shortcomings of the AAOP version of the AOPA's cost accounting system in terms of inequity between disciplines and between procedures within the same discipline. This understanding was achieved through an exhaustive demonstration comparing the cost of procedures under the current formula with the cost of procedures under Dr. Jacobs' proposed formulas. The report by Framework Partners Inc, presented to a meeting of AAOP by Mr. Gord Allen, MBA, (September 19, 2002) validated Dr. Jacobs' earlier conclusions and further reported that that this initial discount made has subsequently resulted in a differential in the effective hourly rate between prosthetics and orthotics that grew from 14.54% in 1991 to 39.75% in 2002.

Framework Partners successfully surveyed twenty-seven out of a total of 28 facilities in Alberta, for times to perform procedures for every procedure in the AADL Approved Product Lists - orthotics and prosthetics. The times to perform procedures were weighted based on the actual volume of procedures performed by each provider in the 2000-2001 AADL benefit year. Working groups of Clinicians in Edmonton and Calgary reviewed and verified the average weighted times to perform every procedure. The new AADL Approved Product Lists - orthotics and prosthetics were presented in compliance with global budget revenue neutrality for 2000-2001, as required by AADL who generously contributed valuable consumption data to the exercise.

Contrary to Dr. Jacobs' recommendation for two separate formulas, and demonstrated in the Framework report, AAOP voted for a single, blended rate. Separate rates for prosthetics and orthotics, within global budget neutrality, involve simply reallocating the mark up on components in orthotics to the labour rate for a service model of pricing for orthotics. Blending the rate for prosthetics with the rate for orthotics retained the retail model of pricing.

To the extent that the new retail oriented formula was based on thoroughly up-to-date times to perform procedures as of 2002, the formula implemented by AADL in 2003 represented an improvement over the previous retail model. While markups were not eliminated, they were corrected to at least narrow the differential in the effective hourly rate between prosthetics and orthotics:


Price=(direct materials×markup)+shipping+(rate×time)

The markup on components has increased since 2003, exclusive of a constant factor for rework, loss and handling charges, maintaining the retail model of pricing and perpetuating the inequity for Orthotics.

Given that all prosthetists and orthotists have the same educational qualifications and must meet the same national standards for certification set out by the Orthotics Prosthetics Canada, orthotists deserve equal pay for work of equal value.

### Governance of AAOP

In recognition of the findings of Dr. Jacob's research later validated by Framework, AADL has on more than one occasion offered to implement separate applications of the AOPA Cost Accounting System for Prosthetics (i.e. retail model of pricing based on markup on components) and Orthotics (i.e. service model of pricing based on times to perform procedures). AAOP has declined these offers.

In order to understand AAOP's position with respect to maintaining a retail model for both disciplines, it may be helpful to review the Bylaws of the AAOP. According to its Bylaws AAOP is loosely constituted as a professional association representing individuals (i.e. Clinicians, Technicians and Associates). It is not a trade association with any authority to represent businesses (i.e. AADL Approved Prosthetic and Orthotic Suppliers). Since the combined number of individual members of prosthetists, prosthetists-orthotists and related technicians and associates exceeds that of individual orthotists and related technicians and associates in AAOP, the prosthetic issues tend to drive the agenda. Hence, the outcome of the AAOP's vote in favour of a single, blended rate in 2003 may reflect a bias.

The aging of the baby boom population is a very well established demographic trend. This trend has created increased demand for orthotic services while simultaneously decreasing the supply of orthotists available to deliver these services as they retire and are not sufficiently replenished. According to the demographic study conducted by the Canadian Association of Prosthetists and Orthotists in 2011, 60% of clinicians at that time were aged 45 and older and, planning to retire between 2021 and 2030.^4^ The majority of these clinicians represented small, owner-managed practices. Despite various studies,^4,10^ identifying the looming shortage of orthotists institutional inertia has failed to address the increased demand for Orthotists let alone met the other educational goal of graduate and post-graduate degrees to provide unbiased, peer-reviewed expertise. The economic impact of a lack of skilled orthotists creates challenges of succession planning for owner-managed practices to transition the knowledge, skills and experience required to maintain sustainable access to orthotic care. The net effect is that fewer and fewer older Orthotists are focusing their practices on smaller and more specialized segments of the market. In particular, lower limb and spinal orthoses.

The governance practices of AAOP also appear to have limited orthotists in other ways. The Alberta Government offered to include prosthetists and orthotists under the Health Professions Act in 1996,^6^ on the condition of licensure as the minimum standard in Allied Health. Again, AAOP declined the offer from the Alberta Government. Prosthetists and orthotists remain unlicensed in Alberta (and every other jurisdiction in Canada). Certification is limited to public education. Licensure encompasses public education and extends beyond that to include public protection. Licensure is a pre-requisite to applying for access to Alberta Netcare, a valuable tool providing fast, secure and confidential access to provincial Electronic Health Records enabling communication between providers all along the patient's continuum of care.

### Merle Taylor Formula: Public sector sets the standard for the private sector

Total compensation for clinicians and technicians employed in the public sector is significantly higher than their counterparts employed in the private sector. Data for the public sector is derived directly from the Collective Agreements^11^ between the Health Sciences Association of Alberta (HSAA) and Alberta Health Services (AHS). Data for the private sector is based from Occupations in Alberta.

Total compensation for clinicians in the public sector is currently $136,666.76 which is $31,859.52 or 23.3% more than in private sector (**Table 1**). Total compensation for technicians in the public sector is currently $113,442.16 which is $43,166.15 or 39.6% more than in the private sector (**Table 2**).

**Table 1:** Comparison of total compensation for clinicians in the public and private sectors.

	Public Sector	Private Sector	Public Sector Premium	
**Salary**				
Hourly	$54.46	$48.26	$6.20	11.4%
Annually	$113,275.95	$100,378.99	$12,896.96	11.4%
**Benefits**				
Mandatory	$4,470.81	$4,428.25	$42.56	1.0%
Non-Mandatory	$18,920.00	n/a	$18,920.00	100.0%
**Total Compensation**	**$136,666.76**	**$104,807.24**	**$31,859.52**	**23.3%**

**Table 2:** Comparison of Total Compensation for Technicians in the Public and Private Sectors.

	Public Sector	Private Sector	Public Sector Premium	
**Salary**				
Hourly	$44.75	$31.71	$13.04	29.0%
Annually	$93,080.00	$65,961.34	$27,118.66	29.0%
**Benefits**				
Mandatory	$4,404.16	$4,314.67	$89,49	2.0%
Non-Mandatory	$15,958.00	n/a	$15,958.00	100.0%
**Total Compensation**	**$113,442.16**	**$70,276.01**	**$43,166.15**	**39.6%**

In recognition of the public sector premium, the Review of Orthotist and Prosthetics Business Arrangements by Merle Taylor Management Consultants commissioned by AADL recommended to a meeting of AAOP (June 10, 2008) that: “*Annual increases to the labour rate should be tied to the public sector P&O labour rate increases*.”

AADL adopted the public sector as the standard for the private sector in 2008 based on total compensation for senior clinicians and technicians defined as step 9 under the collective agreement. Equity with the public sector has been achieved only once, in 2012. Since then, clinicians and technicians in the private sector have not received equal pay for work of equal value compared with their counterparts in the public sector.

Given that all prosthetists and orthotists employed in the private and public sectors are required to have the same educational qualifications and must meet the same national standards for certification as set out by the orthotics prosthetics Canada, clinicians and technicians employed in the private and public sectors deserve equal pay for work of equal value.

### CALL TO ACTION

Prosthetics and orthotics in Alberta is characterized by long-standing inequities between disciplines and, between public and private sectors. These inequities have had a serious negative impact on sustainable access to orthotic care for Albertans with chronic conditions.

The inequity between orthotics and prosthetics is structural. It was imbedded into AAOP's version of the AOPA cost accounting methodology in 1991 when AAOP discounted times to perform orthotic procedures by 20% and added a novel markup on components. This retail model of pricing has benefited prosthetics at the expense of orthotics for 20 years. Despite AADL offers to maintain the existing retail model for prosthetics while creating a separate service model for orthotics, as recommended by independent researchers, AAOP has maintained the status quo.

The inequity between the public and private sectors is the direct result of the public sector premium in Alberta, currently 23.3% for clinicians and 39.6% for technicians, and, AADL's failure to implement and maintain the Merle Taylor formula correctly based on total compensation in the public sector.

Sustainable access to orthotic care for Albertans depends on AADL, as the policy maker, working together with prosthetic and orthotic suppliers, to demonstrate their leadership to resolve these inequities. It is recommended that AADL build on their previous research by Dr. Philip Jacobs, independently validated by Framework Partners Inc., to:

1. Engage a qualified consultant to implement and maintain a service model of pricing for orthotic procedures based on times to perform procedures according to the Merle Taylor formula including:
  a) The rate must be based on total compensation in the public sector derived directly from the Collective Agreement between the HSAA and AHS.
  b) A rigorous pricing review adjustment process including manufacturers suggested retail price provided directly by suppliers as the standard for pricing of components. Consistent sourcing of cost data direct from Suppliers will enable AADL to align prosthetics and orthotics with current practices in other benefit areas.
2. Resolve the shortage of skilled orthotists by amending AADL policy OP-05: specialty assessors for prosthetic and orthotic benefits to recognize foreign trained graduates of the International Prosthetics and Orthotics Society's (ISPO) category one programs as eligible to practice in Alberta. In the medium to longer-term, explore opportunities to address the educational void by developing a graduate program in collaboration with post-secondary institutions in Alberta with programs in kinesiology, biomedical engineering, physical medicine and rehabilitation, rehabilitation engineering and assistive technologies.
3. Bring prosthetics and orthotics into alignment with the standard of allied health professions under the health professions act to enable them access to electronic health records under Alberta Netcare.
4. Improve the efficiency and productivity of orthotists by reducing paper burden. Replace the cumbersome authorizations and claims process for services to existing devices with an adjustment to the cost of the device to include the cost of support and service of the device, particularly for clients aged 18 and under.

## DECLARATION OF CONFLICTING INTERESTS

As a key member, AADL agreements teams (2000 – 2013) including chair, AAOP steering committee – study of formula and fee schedule (2000–2003), Nancy has developed in-depth knowledge and experience with the economics of pricing of Orthotics and Prosthetics in Alberta including the granular data and pricing models. She has made a significant contribution to ensuring sustainable access to prosthetic and orthotic care for Albertans with disabilities. She successfully initiated the transition from a retail to a professional services model of pricing for orthotics, created generic codes to dramatically simplify fee schedules, and effectively changed the landscape to attract investment in biomechanical sciences and engineering research required to advance clinical practice.

## SOURCES OF SUPPORT

Braceworks benefits from federal and provincial support under BioTalent Canada, MiTacs and IRAP to provide opportunities for young undergraduate and graduate students in Biomedical Engineering from the University of Calgary, University of Waterloo, University of British Columbia and Simon Fraser University to contribute to advancing The Calgary Protocol.

## AUTHOR SCIENTIFIC BIOGRAPHY

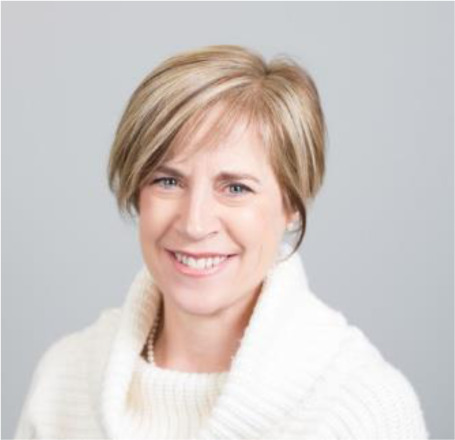



Braceworks is distinguished by expertise in pediatric clinical practice supported by full-time, professional management and product planning. Nancy Schneider BCom, Co-founder and CEO since 1996 is the operational manager leading a high performance team delivering capacity utilization rates that consistently exceed the industry standards while successfully diversifying into product development through applied research.
